# Independent Prognostic Significance of Perforation in Colorectal Cancer: Insights From a Propensity Score‐Matched Cohort Study

**DOI:** 10.1002/ags3.70163

**Published:** 2025-12-29

**Authors:** Yoshiaki Fujii, Shuhei Ueno, Yoshinaga Aoyama, Hiroyuki Imafuji, Takahisa Hirokawa, Hirotaka Miyai, Masahiro Kimura, Kenji Kobayashi, Shuji Takiguchi

**Affiliations:** ^1^ Department of Surgery Kariya Toyota General Hospital Kariya Japan; ^2^ Department of Gastroenterological Surgery Nagoya City University Graduate School of Medical Sciences Nagoya Japan

**Keywords:** colorectal neoplasms, oncologic outcome, overall survival, perforation, prognostic factor

## Abstract

**Aim:**

Although perforated colorectal cancer has a poor prognosis, it remains unclear whether this is attributed to the perforation itself or related clinicopathological factors indicating advanced tumor progression. We investigated the true prognostic impact of perforation in colorectal cancer after adjusting for perforation‐related clinicopathological factors.

**Methods:**

This retrospective single‐center cohort study included 2073 patients underwent curative resection for primary colorectal cancer between January 2009 and December 2022 (73 with perforated colorectal cancer and 2000 with nonperforated colorectal cancer). Perforation‐related clinicopathological factors were identified by multivariate logistic regression and used for 1:2 propensity score matching as covariates. Recurrence‐free survival and overall survival were analyzed using Kaplan–Meier estimates and Cox proportional hazards models.

**Results:**

Independent perforation‐related clinicopathological factors were age ≥ 75 years, left‐sided tumor, and pathological T Stage 4 (pT4). The perforated colorectal cancer group had a lower rate of minimally invasive surgery (62.5% vs. 90.6%), fewer harvested lymph nodes (medians: 13 vs. 16.5), and more frequent major postoperative complications (18.8% vs. 7.3%) compared to the nonperforated colorectal cancer group. Multivariate Cox analysis revealed that pT4 and perforation independently predicted worse recurrence‐free survival and overall survival. The hazard ratios were 2.71 and 2.54 for pT4 (95% confidence intervals: 1.45–5.04 and 1.08–5.9), and 2.67 and 3.30 for perforation (95% confidence intervals: 1.44–4.96 and 1.37–7.96), respectively.

**Conclusion:**

Perforation independently predicted poor prognosis in colorectal cancer, highlighting its distinct oncologic relevance beyond standard pathological staging. Prospective studies should establish individualized strategies to improve outcomes in perforated colorectal cancer.

## Graphical abstract

Perforated colorectal cancer (PCC) is considered to have a poor prognosis; however, it remains unclear whether this is attributable to perforation itself or to perforation‐related clinicopathological factors. In this study, we analyzed prognosis using propensity score matching with perforation‐related factors and demonstrated that perforation is an independent adverse prognostic factor for both recurrence‐free and overall survival. Thus, PCC has a distinct prognostic significance beyond conventional pathological staging.

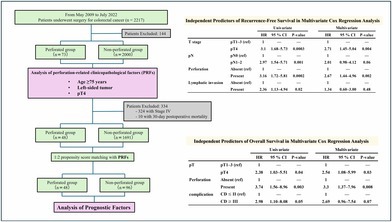

## 1 Introduction

Perforated colorectal cancer (PCC) is uncommon, occurring in approximately 2%–10% of colorectal cancer (CRC) cases and usually requiring urgent surgery [1]. Previous studies have reported that perforation‐related clinicopathological factors (PRFs) are associated with male sex, mucinous histology, T4 tumors, N2 lymph node metastasis, and Stage IV CRCs [2, 3]. However, these studies were primarily based on univariate analyses, and no studies have investigated PRFs after adjusting for other relevant clinicopathological factors.

On the other hand, PCC reportedly worsens short‐term outcomes and long‐term prognoses [3, 4, 5]. Regarding short‐term prognoses, the 30‐day postoperative mortality rate of patients with PCC is 4.5%–17% due to severe complications, including peritonitis, sepsis, and organ failure, establishing perforation as a risk factor for early postoperative death [2, 3, 4, 6, 7, 8, 9, 10, 11, 12, 13, 14, 15]. Regarding long‐term prognoses, patients with PCC have significantly worse overall survival (OS) and recurrence‐free survival (RFS) rates compared to those with non‐PCC (NPCC) [3, 6, 8, 9, 11, 16]. Clinicopathological factors, such as male sex, lymph node metastasis, lymphatic invasion, left‐sided tumor location, pathological T Stage 4 (pT4), and a lower number of harvested lymph nodes, have been identified as independent prognostic factors for adverse long‐term outcomes [3, 7, 8, 10, 13, 15]. Importantly, perforation is an independent prognostic factor in many studies [3, 8, 15, 17].

However, most previous studies did not adjust for PRFs, which are crucial factors that should be considered in evaluating the prognostic relevance of perforation. Consequently, it remains unclear whether the poor outcomes observed in PCC stem from the perforation itself or merely reflect the adverse influence of PRFs. This ambiguity raises the concern that the effect of perforation may have been either overestimated or underestimated. Indeed, several reports have suggested that perforation is not an independent prognostic determinant in PCC [9, 11, 14]. To date, no studies have explicitly examined whether perforation has intrinsic prognostic significance or simply mirrors the impact of PRFs.

In this study, we aimed to (1) identify PRFs associated with the occurrence of perforation and (2) determine the true independent prognostic impact of perforation. To achieve this, propensity score matching (PSM) was performed using the identified PRFs as covariates to balance background characteristics between the PCC and NPCC groups. Our findings may clarify the oncologic relevance of perforation and contribute to more evidence‐based, individualized management strategies for patients with PCC.

## 2 Methods

### 2.1 Study Design and Patient Selection

This single‐center retrospective cohort study included 2217 patients who underwent surgery for Stage I–IV primary colorectal cancer at Kariya Toyota General Hospital between January 2009 and December 2022. The study protocol was approved by the Institutional Review Board of Kariya Toyota General Hospital (Approval No. 1149) and was conducted according to the ethical principles of the Declaration of Helsinki. The requirement for informed consent was waived due to the retrospective nature of the study and the use of anonymized data.

Patients were excluded if they had incomplete data, iatrogenic colorectal perforation, noncurative resection, Tis lesions, received neoadjuvant therapy, or were diagnosed with appendiceal cancer. Most missing data involved key clinicopathological factors (e.g., T/N/M stage or outcomes). No imputation was performed, and the impact on results is considered negligible. PCC was defined as tumor‐related colonic wall disruption with leakage or abscess formation and diagnosed based on computed tomography (CT), intraoperative findings, surgical records, and pathology. All patients with PCC required urgent or semi‐urgent surgery; no elective procedures were performed. Perforation was considered tumor‐related when anatomically contiguous with or adjacent to the tumor. We defined the right‐sided colon as the cecum to the transverse colon, and the left‐sided colorectum as the descending colon to the upper rectum. Tumor obstruction was defined as decompression‐requiring symptoms and radiological findings. TNM classification followed the 8th edition of the UICC system [18].

### 2.2 Identification of PRFs

To identify PRFs, univariate and multivariate logistic regression analyses were used to compare the PCC and NPCC groups. Explanatory variables included age (≥ 75 vs. < 75 years), sex, tumor location (right‐sided vs. left‐sided), pT stage (pT4 vs. pT1–3), pathological N (pN) stage (pN0 vs. pN1–2), M stage (M0 vs. M1), lymphatic invasion, vascular invasion, the presence of mucinous or poorly differentiated histological components, and tumor obstruction. Significant variables in the multivariate analysis were defined as PRFs.

### 2.3 PSM and Prognostic Analysis

For prognostic analysis, patients with Stage IV and those who died within 30 days postoperatively were excluded. Among the remaining patients, a 1:2 PSM was performed using previously identified PRFs (age, tumor location, and pT stage), with sex included as covariates. Although pT is a postoperative variable, it was included in the propensity score model as a surrogate for baseline tumor burden due to its significant association with perforation. In clinical practice, a definitive diagnosis of cancer is often not established before surgery in patients with PCC. In addition, severe inflammation or abscess formation commonly makes it difficult to accurately evaluate tumor size and depth of invasion on CT. Therefore, the use of pT was considered necessary to achieve appropriate balance in disease severity between groups. In the subsequent analysis of oncologic outcomes, pT was included in the outcome analysis due to its well‐established association with prognosis. Thus, pT served two distinct purposes: first, as a covariate in the propensity score model to balance baseline tumor burden; and second, as a prognostic factor in the multivariate Cox model. This yielded a matched cohort of 48 and 96 patients in the PCC and NPCC groups, respectively. Post‐PSM comparisons included surgical approach (open vs. minimally invasive surgery [MIS]), surgical procedure (resection with anastomosis vs. resection with stoma formation), number of harvested lymph nodes, postoperative complications, and adjuvant chemotherapy (AC), including administration status (present/absent), time to the initiation of AC (in days), and regimen (fluoropyrimidine monotherapy or oxaliplatin‐containing). MIS was defined as laparoscopic or robotic procedures. Postoperative complications were assessed using the Clavien–Dindo (CD) classification and categorized as Grades 0–II (minor) or III–V (major). Recurrence was defined by CT‐detected distant, peritoneal, or local lesions; histological confirmation was not required. The date of the first radiologic detection was used. Death from CRC was treated as an event; other deaths were censored. RFS was defined as the time from surgery to recurrence or cancer‐related death. OS was defined as the time from surgery to death from any cause, with censoring at the last follow‐up. Cox proportional hazard models were used to assess recurrence and survival outcomes. Univariate analysis was performed to evaluate candidate prognostic variables, including perforation status. Variables with a *p*‐value < 0.1 and those deemed clinically significant were included in the multivariate models.

### 2.4 Statistical Analysis

For comparisons between the groups, the Mann–Whitney *U* test was used for continuous variables, and the Chi‐square test was used for categorical variables. Univariate and multivariate logistic regression analyses were performed to identify PRFs. PSM was conducted between the PCC and NPCC groups using PRFs and sex as covariates using a 1:2 nearest neighbor method and a caliper width of 0.2. The adequacy of matching was evaluated using the standardized mean difference (SMD), with SMD < 0.1 indicating good balance. For survival analysis, Kaplan–Meier curves were generated for RFS and OS, which were compared using the log‐rank test. Prognostic factors for RFS and OS were assessed using Cox proportional hazards models, with covariates including perforation status and other clinicopathological variables. All statistical analyses were performed using R software (version 4.3.2, R Foundation for Statistical Computing, Vienna, Austria) and JMP (SAS Institute Inc., Cary, NC, USA). A *p*‐value < 0.05 was considered to indicate statistical significance. In addition to the primary analyses, we performed two sensitivity analyses. First, we performed subgroup analyses restricted to the adjuvant chemotherapy–eligible cohort (ACE cohort). AC eligibility was defined as the presence of any of the following high‐risk features: pT4, perforation, stage III disease, < 12 harvested lymph nodes, poorly differentiated adenocarcinoma, signet‐ring cell carcinoma, and mucinous adenocarcinoma. In the ACE cohort, we evaluated AC, including administration status (present/absent), time to initiation (days), and regimen (fluoropyrimidine monotherapy or oxaliplatin‐containing), as well as initial recurrence patterns. Multivariate Cox regression analyses were then conducted for RFS and OS, and Kaplan–Meier analyses were performed according to AC administration. Second, given the long study period (2009–2022), we conducted an additional analysis to evaluate potential era effects. To account for temporal changes, the year of surgery was categorized into three periods (2009–2013, 2014–2018, and 2019–2022) and incorporated as a covariate in the Cox models. Furthermore, we calculated the hazard ratios (HRs) (with 95% confidence intervals [CIs]) for the prognostic impact of perforation within each period and constructed a forest plot.

## 3 Results

**FIGURE 1:**
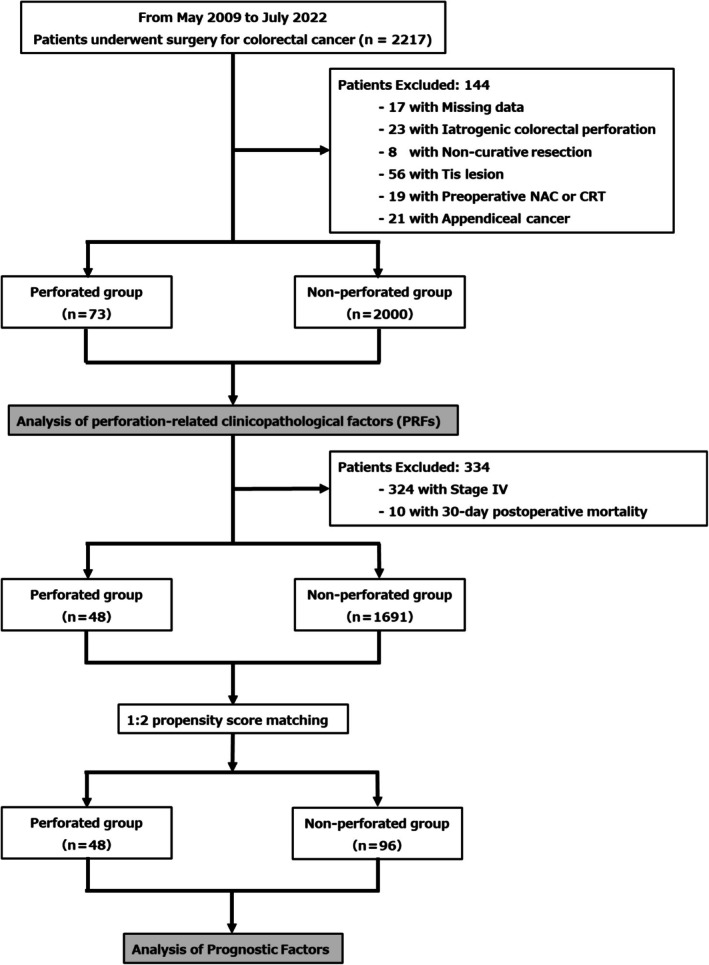
Study flow diagram illustrating the identification of perforation‐related clinicopathological factors and prognostic evaluation in perforated colorectal cancer. The diagram shows patient selection and grouping for the identification of PRFs and subsequent prognostic analysis in PCC using propensity score matching. CRT, chemoradiotherapy; NAC, neoadjuvant chemotherapy; PCC, perforated colorectal cancer; PRFs, perforation‐related clinicopathological factors.

Based on these criteria, patients were classified into the PCC (*n* = 73) or NPCC (*n* = 2000) group (Figure 1). The median follow‐up periods were 57.7 and 49.6 months for OS and RFS, respectively. In a comparison of baseline characteristics, the PCC group showed significantly higher rates of left‐sided tumors, pT4, Stage IV, lymphatic invasion, and vascular invasion than the NPCC group. The 30 day postoperative mortality was higher in the PCC group than in the NPCC group (6.8% vs. 0.2%, *p* < 0.001) (Table 1). Logistic regression analysis was performed to identify PRFs (Table 2). In the univariate analysis, left‐sided tumor location, pT4, M1, lymphatic invasion, and vascular invasion were significantly associated with PCC. These variables, along with the N category and age, were included as covariates in the multivariate model. Age ≥ 75 years (odds ratio [OR]: 1.79, 95% CI: 1.10–2.90, *p =* 0.02), left‐sided tumor location (OR: 2.10, 95% CI: 1.20–3.69, *p =* 0.01), and pT4 (OR: 2.46, 95% CI: 1.45–4.16, *p =* 0.0009) were identified as independent PRFs. To adjust for baseline characteristics associated with the occurrence of perforation between the PCC and NPCC groups, 1:2 PSM was performed using age, tumor location, pT stage (identified as PRFs), and sex, as covariates. Subsequently, 48 patients in the PCC group were matched to 96 in the NPCC group. All covariates achieved an SMD < 0.1, confirming a good balance between the groups (Table 3 and Figure S1).

**TABLE 1:** Clinicopathological characteristics of patients with PCC and NPCC.

	NPCC group (*N* = 2000)	PCC group (*N* = 73)	*p*
**Age**			
≥ 75 years	712 (35.6%)	34 (46.6%)	0.059
< 75 years	1288 (64.4%)	39 (53.4%)	
**Sex**			
Male	1195 (59.8%)	39 (53.4%)	0.28
Female	805 (40.2%)	34 (46.6%)	
**Tumor location**			
Right‐sided	714 (35.7%)	17 (23.3%)	0.02
Left‐sided	1286 (64.3%)	56 (76.7%)	
**pT stage**			
pT1‐3	1614 (80.7%)	43 (58.9%)	< 0.001
pT4	386 (19.3%)	30 (41.1%)	
**pN stage**			
pN0	1163 (58.2%)	35 (47.9%)	0.09
pN1‐2	837 (41.8%)	38 (52.1%)	
**M stage**			
M0	1696 (84.8%)	53 (72.6%)	0.009
M1	304 (15.2%)	20 (27.4%)	
**Stage**			
I	449 (22.5%)	1 (1.4%)	< 0.001
II	646 (32.3%)	24 (32.9%)	
III	601 (30.0%)	28 (36.9%)	
IV	304 (15.2%)	20 (28.8%)	
**Lymphatic invasion**			
Absent	801 (40.1%)	18 (24.7%)	0.006
Present	1199 (59.9%)	55 (75.3%)	
**Vascular invasion**			
Absent	1478 (73.9%)	46 (63.0%)	0.045
Present	522 (26.1%)	27 (37.0%)	
**Por/muc histology**			
Absent	1772 (88.6%)	61 (83.6%)	0.2
Present	228 (11.4%)	12 (16.4%)	
**Tumor obstruction**			
Absent	1838 (91.9%)	63 (86.3%)	0.12
Present	162 (8.1%)	10 (13.7%)	
**30‐day mortality**	5 (0.2%)	5 (6.8%)	< 0.001

*Note:* Values are presented as number (%).

Abbreviations: NPCC, nonperforated colorectal cancer; PCC, perforated colorectal cancer.

**TABLE 2:** Univariate and multivariate logistic regression analyses to identify PRFs in colorectal cancer.

	Univariate			Multivariate		
	OR	95% CI	*p*	OR	95% CI	*p*
**Age**						
< 75 years (Ref)	1			1		
≥ 75 years	1.58	0.99–2.52	0.057	1.79	1.10–2.90	0.02
**Tumor location**						
Right‐sided (Ref)	1			1		
Left‐sided	1.83	1.05–3.17	0.03	2.1	1.2–3.69	0.01
**T stage**						
pT1–3 (Ref)	1			1		
pT4	2.92	1.81–4.71	< 0.001	2.46	1.45–4.16	0.0009
**N stage**						
pN0 (Ref)	1			1		
pN1–2	1.51	0.95–2.41	0.09	0.96	0.56–1.65	0.88
**M stage**						
M0 (Ref)	1			1		
M1	2.111	1.24–3.57	0.006	1.37	0.75–2.50	0.31
**Sex**						
Male (Ref)	1					
Female	1.29	0.81–2.07	0.28			
**Lymphatic invasion**						
Absent (Ref)	1			1		
Present	2.04	1.19–3.50	0.01	1.66	0.90–3.05	0.11
**Vascular invasion**						
Absent (Ref)	1			1		
Present	1.66	1.02–2.70	0.04	1.08	0.63–1.85	0.77
**Por/muc histology**						
Absent (Ref)	1					
Present	1.53	0.81–2.88	0.19			
**Tumor obstruction**						
Absent (Ref)	1					
Present	1.8	0.91–3.58	0.12			

Abbreviations: CI, confidence interval; OR, odds ratio; PRFs, perforation‐related factors; Ref, reference category.

**TABLE 3:** Patient characteristics before and after propensity score matching.

	Before propensity score matching				After propensity score matching			
	PCC (*n* = 48)	NPCC (*n* = 1691)	*p*	SMD	PCC (*n* = 48)	NPCC (*n* = 96)	*p*	SMD
**Age**								
≥ 75 years	26 (54.2%)	603 (35.6%)	0.01	0.38	26 (54.2%)	50 (52.1%)	0.81	0.042
< 75 years	22 (45.8%)	1088 (64.4%)			22 (45.8%)	46 (47.9%)		
**Sex**								
Male	25 (52.1%)	1004 (59.4%)	0.31	0.15	25 (52.1%)	53 (55.2%)	0.72	0.063
Female	23 (47.9%)	687 (40.6%)			23 (47.9%)	43 (44.8%)		
**Tumor location**								
Right‐sided	7 (14.6%)	601 (35.5%)	0.001	0.5	7 (14.6%)	13 (13.5%)	0.87	0.03
Left‐sided	41 (85.4%)	1090 (64.5%)			41 (85.4%)	83 (86.5%)		
**pT stage**								
pT1‐3	31 (64.6%)	1458 (86.2%)	< 0.001	0.52	31 (64.6%)	62 (64.6%)	1	0
pT4	17 (35.4%)	233 (13.8%)			17 (35.4%)	34 (35.4%)		
**pN stage**								
pN0	21 (43.8%)	1093 (64.6%)	0.004	—	21 (43.8%)	56 (58.3%)	0.1	—
pN1‐2	27 (56.3%)	598 (35.4%)			27 (56.3%)	40 (41.7%)		
**Stage**								
I	1 (2.1%)	441 (26.1%)	< 0.001		1 (2.1%)	2 (2.1%)	0.25	
II	20 (41.7%)	623 (36.8%)			20 (41.7%)	54 (56.3%)		
III	27 (56.2%)	627 (37.1%)			27 (56.2%)	40 (41.6%)		
**Lymphatic invasion**								
Absent	14 (29.2%)	750 (44.4%)	0.03	—	14 (29.2%)	38 (39.6%)	0.22	—
Present	34 (70.8%)	941 (55.6%)			34 (70.8%)	58 (60.4%)		
**Vascular invasion**								
Absent	35 (72.9%)	1342 (79.4%)	0.29	—	35 (72.9%)	75 (78.1%)	0.49	—
Present	13 (27.1%)	349 (20.6%)			13 (27.1%)	21 (21.9%)		
**Por/muc histology**								
Absent	42 (87.5%)	1506 (89.0%)	0.74	—	42 (87.5%)	91 (94.8%)	0.13	—
Present	6 (12.5%)	185 (11.0%)			6 (12.5%)	5 (5.2%)		
**Tumor obstruction**								
Absent	47 (97.9%)	1596 (94.3%)	0.23	—	47 (97.9%)	86 (89.6%)	0.05	—
Present	1 (2.1%)	95 (5.7%)			1 (2.1%)	10 (10.4%)		

*Note:* Percentages are shown in parentheses.

Abbreviations: NPCC, nonperforated colorectal cancer; PCC, perforated colorectal cancer; SMD, standardized mean difference.

Table 4 presents a comparison of surgical approaches, surgical procedures, numbers of harvested lymph nodes, postoperative complications, adjuvant chemotherapies, and initial recurrence patterns after PSM. Open surgery and resection with stoma formation were significantly more frequent in the PCC group, whereas MIS and resection with anastomosis predominated in the NPCC group (both *p* < 0.001). The number of harvested lymph nodes was significantly lower in the PCC group (medians: 13 vs. 16.5, *p =* 0.008). Severe postoperative complications (CD Grades III and IV) were significantly more frequent in the PCC group (*p =* 0.046). No significant differences were observed between the groups in the rate of AC, the interval from surgery to chemotherapy initiation, or the use of oxaliplatin‐containing regimens. Regarding initial recurrence patterns, distant metastasis was significantly more frequent in the PCC group than in the NPCC group (*p =* 0.004). Although peritoneal recurrence occurred slightly more often in the PCC group than in the NPCC group, the difference was not statistically significant (*p* = 0.14), and no significant difference was observed in local recurrence between the groups.

**TABLE 4:** Comparison of surgical characteristics, postoperative outcomes, adjuvant therapy, and initial recurrence patterns after matching.

	PCC (*n* = 48)	NPCC (*n* = 96)	*p*
**Surgical approach**			
Open	18 (37.5%)	9 (9.4%)	< 0.001
MIS	30 (62.5%)	87 (90.6%)	
**Surgical procedure**			
Resection with anastomosis	27 (56.3%)	88 (91.7%)	< 0.001
Resection with stoma formation	21 (43.7%)	8 (8.3%)	
**Number of harvested lymph nodes**	13 (6–19)	16.5 (12–25)	0.008
**Postoperative complications**			
Clavien–Dindo grade 0–II	39 (81.3%)	89 (92.7%)	0.046
Clavien–Dindo grade III–IV	9 (18.8%)	7 (7.3%)	
**Adjuvant chemotherapy**			
Received	20 (41.7%)	32 (33.3%)	0.33
Not received	28 (58.3%)	64 (66.7%)	
**Interval to adjuvant chemotherapy (days)**	33.5 (29–46.5)	34 (26–45)	0.6
**Adjuvant chemotherapy regimen**			
Fluoropyrimidine monotherapy	14 (29.2%)	24 (25.0%)	0.69
Oxaliplatin‐containing regimen	6 (12.5%)	8 (8.3%)	
**Initial recurrence pattern**			
Distant metastasis	16 (33.3%)	12 (12.5%)	0.004
Peritoneal dissemination	8 (16.7%)	8 (8.3%)	0.14
Local recurrence	0 (0%)	2 (2.1%)	0.2

*Note:* Data are presented as median (interquartile range) or number (%).

Abbreviations: MIS, minimally invasive surgery; NPCC, nonperforated colorectal cancer; PCC, perforated colorectal cancer.

**FIGURE 2:**
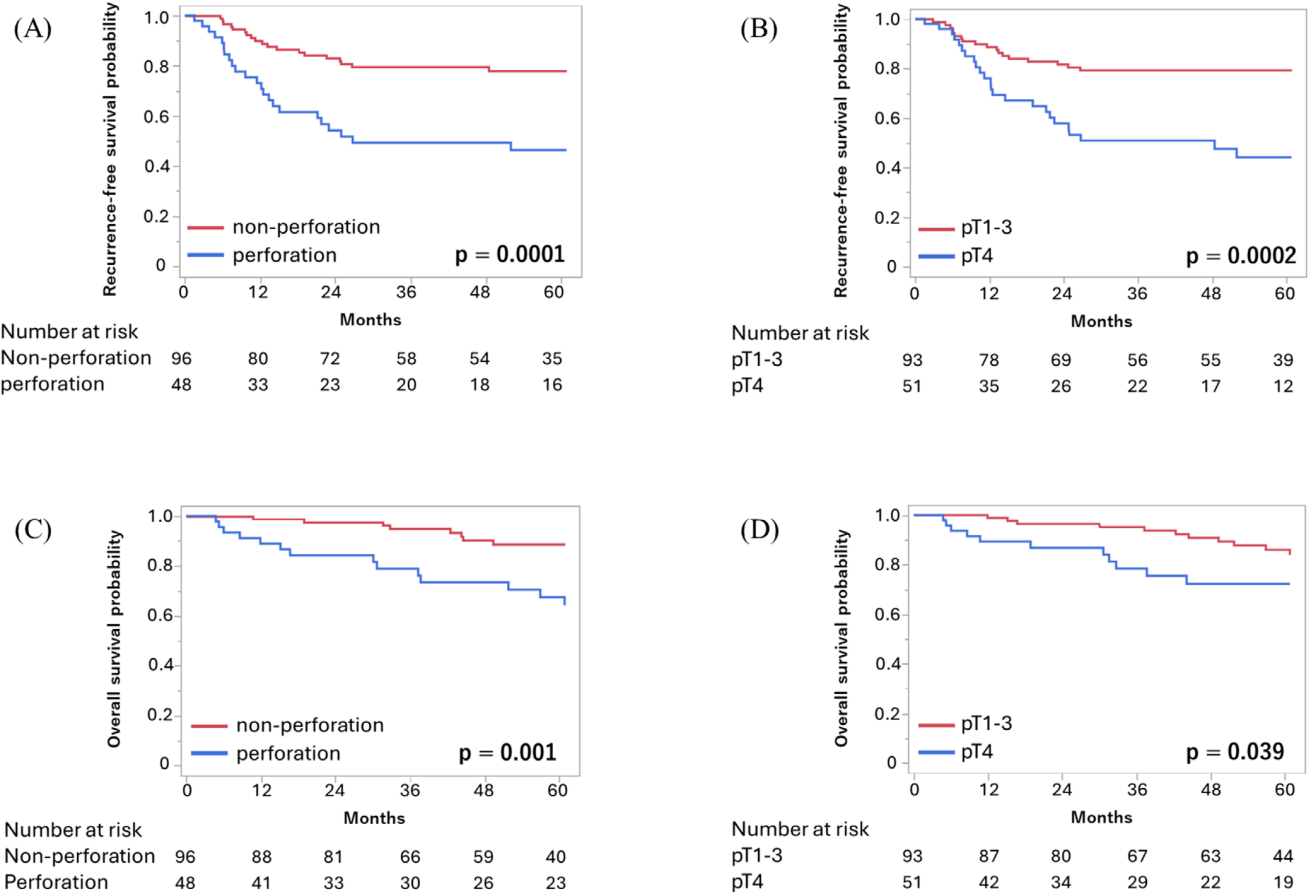
Recurrence‐free survival (RFS) and overall survival (OS) according to the perforation status and pT stage. (A) RFS was significantly shorter in the perforated colorectal cancer group than in the nonperforated colorectal cancer group (log‐rank *p* = 0.0001; 5‐year RFS: 46.4% vs. 78.0%). (B) Patients with pT4 tumors had significantly poorer RFS than those with pT1–3 tumors (log‐rank *p* = 0.0002; 5‐year RFS: 44.1% vs. 67.6%). pT, pathological T stage; RFS, recurrence‐free survival. (C) OS was significantly shorter in the perforated colorectal cancer group than in the nonperforated colorectal cancer group (log‐rank *p* = 0.001; 5‐year OS: 64.2% vs. 88.7%). (D) Patients with pT4 tumors had significantly poorer OS than those with pT1–3 tumors (log‐rank *p* = 0.04; 5‐year OS: 75.3% vs. 83.9%). pT, pathological T stage; OS, overall survival.

Cox proportional hazard models were used to identify prognostic factors. In the univariate analysis for RFS, pT4, pN1–2, perforation, and lymphatic invasion were significantly associated with recurrence. Multivariate analysis revealed pT4 (HR: 2.71, 95% CI: 1.45–5.04, *p =* 0.004) and perforation (HR: 2.67, 95% CI: 1.44–4.96, *p =* 0.002) as independent risk factors for recurrence (Table 5). Although the pN stage was not statistically significant between the groups (*p =* 0.06), a borderline trend was observed. For OS, univariate analysis showed significant associations with pT4, perforation, and CD Grade ≥ III or postoperative complications. In the multivariate model, only pT4 (HR: 2.54, 95% CI: 1.08–5.99, *p =* 0.03) and perforation (HR: 3.3, 95% CI: 1.37–7.96, *p =* 0.008) remained as independent prognostic factors (Table 6). Although CD Grade ≥ III complications were not statistically significant between the groups, a borderline association with OS was observed (*p =* 0.07). AC was not significantly associated with improved RFS or OS (for RFS, HR: 1.65; for OS, HR: 1.35). Kaplan–Meier survival curves demonstrated significantly poorer RFS and OS in the PCC group and patients with pT4 tumors (Figure 2). The 5 year RFS rates were 46.4% for PCC and 78.0% for NPCC, and the 5 year OS rates were 64.2% and 88.7%, respectively. For the pT stage, 5 year RFS and OS rates were 44.1% and 75.3% in pT4, respectively, compared with 67.6% and 83.9% in pT1–3.

**TABLE 5:** Independent predictors of recurrence‐free survival in multivariate Cox regression analysis.

	Univariate			Multivariate		
	HR	95% CI	*p*	HR	95% CI	*p*
**Age**						
< 75 years (ref)	1	—	—	—	—	—
**Sex**						
Female (ref)	1	—	—	—	—	—
**Tumor location**						
Left‐sided (ref)	1	—	—	—	—	—
Right‐sided	1.71	0.79–3.70	0.17	—	—	—
**T stage**						
pT1–3 (ref)	1	—	—	1	—	—
pT4	3.1	1.68–5.73	0.0003	2.71	1.45–5.04	0.004
**pN**						
pN0 (ref)	1	—	—	1	—	—
pN1–2	2.97	1.54–5.71	0.001	2.01	0.98–4.12	0.06
**Perforation**						
Absent (ref)	1	—	—	1	—	—
Present	3.16	1.72–5.81	0.0002	2.67	1.44–4.96	0.002
**Lymphatic invasion**						
Absent (ref)	1	—	—	1	—	—
Present	2.36	1.13–4.94	0.02	1.34	0.60–3.00	0.48
**Vascular invasion**						
Absent (ref)	1	—	—		—	—
Present	1.8	0.079	0.08		—	—
**Por/muc histology**						
Absent (ref)	1	—	—		—	—
Present	1.24	0.44–3.49	0.68		—	—
**Tumor obstruction**						
Absent (ref)	1	—	—		—	—
Present	0.64	0.15–2.65	0.54		—	—
**Adjuvant chemotherapy**						
Not received (ref)	1	—	—		—	—
Received	1.65	0.90–3.02	0.11		—	—
**Postoperative complication**						
CD ≤ II (ref)	1	—	—		—	—
CD ≥ III	1.4	0.55–3.56	0.48		—	—

Abbreviations: CD, Clavien–Dindo classification; CI, confidence interval; HR, hazard ratio; Ref, reference category.

**TABLE 6:** Independent predictors of overall survival in multivariate Cox regression analysis.

	Univariate			Multivariate		
	HR	95% CI	*p*	HR	95% CI	*p*
**Age**						
< 75 years (ref)	1	—	—	—	—	—
≥ 75 years	0.93	0.40–2.15	0.86	—	—	—
**Sex**						
Female (ref)	1	—	—	—	—	—
Male	1.45	0.62–3.40	0.39	—	—	—
**Tumor location**						
Left‐sided (ref)	1	—	—	—	—	—
Right‐sided	2.3	0.85–6.27	0.11	—	—	—
**pT**						
pT1–3 (ref)	1	—	—	1	—	—
pT4	2.38	1.03–5.51	0.04	2.54	1.08–5.99	0.03
**pN**						
pN0 (ref)	1	—	—	—	—	—
pN1–2	1.6	0.68–3.74	0.28	—	—	—
**Perforation**						
Absent (ref)	1	—	—	1	—	—
Present	3.74	1.56–8.96	0.003	3.3	1.37–7.96	0.008
**Lymphatic invasion**						
Absent (ref)	1	—	—	—	—	—
Present	1.98	0.73–5.37	0.18	—	—	—
**Vascular invasion**						
Absent (ref)	1	—	—	—	—	—
Present	2.12	0.89–5.06	0.09	—	—	—
**Por/muc histology**						
Absent (ref)	1	—	—	—	—	—
Present	1.9	0.26–14.2	0.53	—	—	—
**Tumor obstruction**						
Absent (ref)	1	—	—	—	—	—
Present	1.48	0.20–11.0	0.7	—	—	—
**Adjuvant chemotherapy**						
Not received (ref)	1	—	—	—	—	—
Received	1.33	0.58–3.10	0.5	—	—	—
**Postoperative complication**						
CD ≤ II (ref)	1	—	—	1	—	—
CD ≥ III	2.98	1.10–8.08	0.05	2.69	0.96–7.54	0.07

Abbreviations: CD, Clavien–Dindo classification; CI, confidence interval; HR, hazard ratio; Ref, reference category.

### 3.1 Subgroup Analysis in the AC‐Eligible Cohort

In the ACE cohort, no significant differences were observed between the PCC and NPCC groups in the rate of adjuvant chemotherapy administration, the interval to initiation, or the type of regimen (Table S1). In multivariate Cox regression analysis, both pT4 (HR: 2.73, 95% CI: 1.40–5.33, *p* = 0.003) and perforation (HR: 2.43, 95% CI: 1.30–4.54, *p* = 0.005) remained independent adverse prognostic factors for RFS (Table S2). In contrast, for OS, perforation (HR: 2.38, 95% CI: 0.96–5.88, *p* = 0.06) and pT4 (HR: 2.11, 95% CI: 0.86–5.14, *p* = 0.1) did not reach statistical significance but showed a trend toward worse prognosis. Severe postoperative complications (CD ≥ III) were also associated with a trend toward poor OS (HR: 2.66, 95% CI: 0.94–7.50, *p* = 0.065) (Table S3). Furthermore, Kaplan–Meier analyses stratified by AC administration revealed no significant differences in either RFS (*p* = 0.48) or OS (*p* = 0.89) (Figure S2).

### 3.2 Surgical Era‐Adjusted Analysis

In the analysis adjusted for surgical era, perforation remained an independent adverse prognostic factor for both RFS (HR: 2.77, 95% CI: 1.43–5.38, *p* = 0.0026) and OS (HR: 3.36, 95% CI: 1.31–8.54, *p* = 0.011) (Tables S4, S5). Additionally, the HRs for perforation in each surgical era consistently exceeded 1.0, indicating that its adverse prognostic impact persisted across all time periods (Figure S3).

## 4 Discussion

Poor prognosis in patients with PCC has been reported in several studies [3, 4, 5, 7, 8, 11, 13, 14, 15, 16]; however, the prognostic significance of perforation as an independent factor remains controversial [9, 11, 14]. Among previous studies, few have adjusted for confounding factors in evaluating PRFs, and none have assessed the true prognostic impact of perforation after accounting for these factors [2, 3, 5, 6, 7, 8, 9, 10, 11, 12, 13, 14, 15, 16, 17]. Especially, the independent prognostic significance of perforation, after minimizing the influence of background factors, remains unclear.

In this study, the identified PRFs were older age (≥ 75 years), left‐sided tumor location, and pT4. These factors may be associated with the development of perforation due to a combination of age‐related structural changes in colonic tissue [19], inflammation, and necrosis caused by local tumor invasion, and mechanical stress from solid fecal content in the left‐sided colorectum. The PCC group had a 30 day mortality of 6.8%, consistent with previous reports [2, 3, 4, 6, 7, 8, 9, 10, 11, 12, 13, 14, 15]. Following PSM, the PCC group had significantly more frequent postoperative complications of CD Grade ≥ III than the NPCC group. These complications are attributable to severe inflammatory responses, such as peritonitis and sepsis, which are caused by perforation and may reflect the perioperative management challenges unique to PCC.

In the prognostic factor analysis, perforation independently predicted poor prognosis and this may be attributed to two possible mechanisms.

First, surgical quality may influence long‐term outcomes. The PCC group had a higher proportion of open surgeries, and MIS was more challenging. Additionally, more patients underwent stoma formation rather than anastomosis in the PCC group. These findings likely reflect poor intraoperative general conditions, local inflammation, adhesions, and the spillage of bowel contents caused by perforation, which increased the technical difficulty and may preclude safe reconstruction. Under such conditions, performing standard lymphadenectomy is challenging. In this study, the PCC group had significantly fewer harvested lymph nodes than the NPCC group, consistent with previous reports [8, 15].

Second, perforation‐associated inflammatory responses may have led to distant metastasis. Proinflammatory cytokines, such as interleukin‐6, tumor necrosis factor‐α, and interleukin‐1β, can enhance the invasiveness of tumor cells and promote metastasis to distant organs, including the lungs and liver [20, 21]. Elevated levels of systemic inflammatory markers, such as C‐reactive protein and the neutrophil‐to‐lymphocyte ratio, are associated with recurrence and poor survival [22, 23]. When perforation occurs, the leakage of bowel contents into the peritoneal cavity may trigger a strong inflammatory response, leading to rapid cytokine release, increased vascular permeability, and changes in the immune microenvironment, enhancing the risk of hematogenous metastasis. In this study, the PCC group had more frequent distant metastasis (the initial recurrence pattern) than the NPCC group, suggesting the involvement of inflammation‐driven metastatic progression.

Perforation is a high‐risk feature warranting oxaliplatin‐based AC in Stage II CRC according to major guidelines, including those of Japan, National Comprehensive Cancer Network, American Society of Clinical Oncology, and European Society for Medical Oncology [24, 25, 26, 27]. However, no significant differences were observed between PCC and NPCC in terms of AC administration rate, time to initiation, or regimen. Similarly, no differences were found between the two groups within the ACE cohort. These findings are considered to mainly reflect the limitations of sample size, changes in the indications for AC due to guideline revisions during the study period, and the heterogeneity of clinical backgrounds. In contrast, in the subgroup analysis restricted to the ACE cohort, perforation remained an independent adverse prognostic factor for RFS, and although not statistically significant for OS, a trend toward poor prognosis was observed. Furthermore, Kaplan–Meier analyses revealed no clear survival benefit of AC in RFS or OS. These results suggest that the adverse prognostic impact of perforation may outweigh the potential survival benefit of AC, indicating that the effect of current AC on improving prognosis in PCC is limited. Therefore, the development of PCC‐specific treatment strategies, including optimized regimen selection and treatment intensity, is warranted. Beyond adjuvant therapy, we also examined whether temporal changes influenced the prognostic impact of perforation. The study period spanned from 2009 to 2022, during which treatment strategies—including perioperative management, guideline recommendations, and the adoption of MIS—evolved substantially. However, even after adjusting for surgical era, Perforation consistently demonstrated a trend toward poorer prognosis for both RFS and OS across all time periods. These findings indicate that the adverse prognostic impact of perforation has not been overcome, even in contemporary clinical practice.

Furthermore, in this study, pT4 was identified as a PRF and an independent poor prognostic factor, alongside perforation. Several previous studies have reported a higher incidence of peritoneal recurrence in patients with PCC [8, 11]; however, in our study, no significant difference was observed in the frequency of peritoneal dissemination as the initial recurrence pattern between the two groups. This discrepancy may be attributed to the strong influence of pT4 as a risk factor for peritoneal recurrence [28, 29, 30], which previous studies may not have adequately adjusted for. These findings may support the dual significance of pT4 in contributing to perforation and tumor aggressiveness.

To our knowledge, this is the first study to demonstrate that PRFs are strongly associated with perforation in CRC, as well as to evaluate the independent prognostic impact of perforation after adjusting for these factors. Similarly, the novelty of this study lies in its use of PSM with PRFs as covariates to adjust for baseline differences between PCC and NPCC, enabling a rigorous evaluation of the independent prognostic impact of colorectal cancer perforation.

This study had several limitations. First, it was a retrospective analysis conducted at a single institution, which may have introduced selection and treatment biases. Moreover, the relatively small sample size, particularly in the PCC group, may have limited the statistical power, leading to borderline associations with prognosis in the multivariate analysis, such as pN stage for RFS and CD Grade ≥ III postoperative complications for OS. Although not statistically significant, these findings might represent clinically relevant effects.

Second, although PSM was used to adjust for background characteristics, residual confounding cannot be eliminated. Important variables, such as tumor molecular features (e.g., *Ras/BRAF* mutations and MSI status), socioeconomic factors, and patient decision‐making processes, were not included in the analysis, which could have influenced prognosis and treatment selection. In addition, pT was included as a covariate in the propensity score model to adjust for tumor burden. However, this decision warrants careful consideration. While pT is a robust indicator of tumor invasion and closely associated with perforation, it is a postoperative variable that may be influenced by inflammatory changes or necrosis caused by perforation itself. Therefore, pT may function as a potential intermediate variable in the causal pathway between perforation and prognosis, introducing the risk of overadjustment bias. We carefully considered tumor‐related factors such as tumor size and depth of invasion on preoperative imaging as potential indicators of tumor extent or burden. However, in many cases in this study, a definitive diagnosis of cancer had not been established before surgery, and the presence of inflammation or abscess made it difficult to accurately assess the local tumor extent preoperatively. Given these uncertainties and the limitations in data reliability, we ultimately selected pT as a covariate, as it could serve as a surrogate indicator reflecting the degree of tumor progression. To overcome these limitations, the incorporation of preoperatively assessable surrogate markers and molecular profiles will be essential in future studies.

Third, in both the PCC and NPCC groups, the implementation rates of AC were limited and possibly influenced by institutional policies and individual patient backgrounds. Moreover, the treatment regimens and intensities varied. Such treatment heterogeneity may have affected the evaluation of AC efficiency. In addition, detailed reasons for nonreceipt of AC were not consistently collected in this retrospective dataset and therefore could not be sufficiently analyzed. This should be considered an important limitation when interpreting the results.

Fourth, in this study, all patients with PCC required urgent or semi‐urgent surgery, and no elective procedures were performed. Some patients with NPCC also underwent semi‐urgent surgery, but the indications were inconsistent, making evaluation difficult. Therefore, analyses according to surgical urgency were not feasible. Furthermore, detailed data on the presence and severity of peritonitis (e.g., localized abscess versus generalized peritonitis) were not sufficiently collected and could not be included in the analyses. Because these factors may influence postoperative complications and long‐term outcomes, they should be considered limitations when interpreting the results of this study.

Finally, this study did not classify whether the perforation occurred within the tumor itself or in the adjacent bowel segment. If this information had been available, it might have allowed for distinction between different mechanisms of perforation—such as direct tumor invasion versus inflammation or ischemia—and enabled a more detailed evaluation of the associated pathological features and prognostic impact. As the clinical implications may differ depending on the underlying mechanism, further investigation is warranted to clarify this issue.

In conclusion, this study is the first to rigorously demonstrate that perforation independently predicts poor prognosis in colorectal cancer after adjustment for PRFs. These findings underscore that perforation is not merely a manifestation of tumor progression but an independent prognostic factor in colorectal cancer, highlighting the importance of individualized management strategies. In particular, careful postoperative assessment of the patient's general condition is essential to individualize and optimize both the selection and timing of AC regimens. In addition, enhanced surveillance for the early detection of recurrence is warranted. This study's findings may support such efforts and contribute to the advancement of personalized medicine for this high‐risk population.

## Author Contributions

**Yoshiaki Fujii:** data curation, conceptualization, methodology, investigation, writing – original draft. **Shuhei Ueno:** software. **Yoshinaga Aoyama:** formal analysis. **Hiroyuki Imafuji:** methodology, formal analysis. **Takahisa Hirokawa:** validation, methodology. **Hirotaka Miyai:** visualization, formal analysis, software. **Masahiro Kimura:** validation, supervision. **Kenji Kobayashi:** validation, visualization, resources. **Shuji Takiguchi:** project administration, supervision.

## Funding

The authors have nothing to report.

## Ethics Statement

This retrospective study was approved by the Institutional Review Board of Kariya Toyota General Hospital (Approval number: 1149). This study was conducted in accordance with the principles of the Declaration of Helsinki.

## Consent

The authors have nothing to report.

## Conflicts of Interest

Dr. Shuji Takiguchi is an Editorial Board Member of Annals of Gastroenterological Surgery. Except for this, the authors declare no conflicts of interest.

## Supporting information


**Figure S1:** Covariate balance before and after propensity score matching. Absolute mean differences in baseline characteristics between the perforated colorectal cancer and nonperforated colorectal cancer groups are shown before (red) and after (blue) PSM. The vertical dashed line represents a standardized difference of 0.1, indicating an acceptable balance. pT, pathological T stage; PSM, propensity score matching.

**Figure S2:** Recurrence‐free survival (RFS) and overall survival (OS) according to adjuvant chemotherapy (AC) status in the AC‐eligible cohort.
(A) RFS did not differ significantly between patients who received AC and those who did not (log‐rank *p* = 0.48). (B) OS likewise showed no significant difference between the groups (log‐rank *p* = 0.89).

**Figure S3:** Hazard ratios (HRs) for recurrence‐free survival (RFS) and overall survival (OS) associated with perforation, stratified by surgical era. Cox proportional hazards models were fitted separately for each era (2009–2013, 2014–2018, and 2019–2022) to estimate HRs associated with perforation. Dots represent HRs, and horizontal lines denote 95% confidence intervals. The vertical dashed line indicates no effect (HR = 1).

**Table S1:** Adjuvant Chemotherapy (AC) administration in the AC‐eligible cohort.

**Table S2:** Independent predictors of recurrence‐free survival in Multivariate Cox regression analysis in the AC‐eligible cohort.

**Table S3:** Independent predictors of overall survival in Multivariate Cox regression analysis in the AC‐eligible cohort.

**Table S4:** Multivariate Cox regression analysis for recurrence‐free survival adjusted for Surgical Era.

**Table S5:** Multivariate Cox regression analysis for overall survival adjusted for Surgical Era.
