# Supplementary figures and images for: Independent Prognostic Significance of Perforation in Colorectal Cancer: Insights From a Propensity Score‐Matched Cohort Study
Source: Ann Gastroenterol Surg. 2025 Dec 29;10(3):779–91. doi: 10.1002/ags3.70163 (PMC13178268; doi:10.1002/ags3.70163)

Supplementary Figure. 1 Covariate balance before and after propensity score matching (PSM)


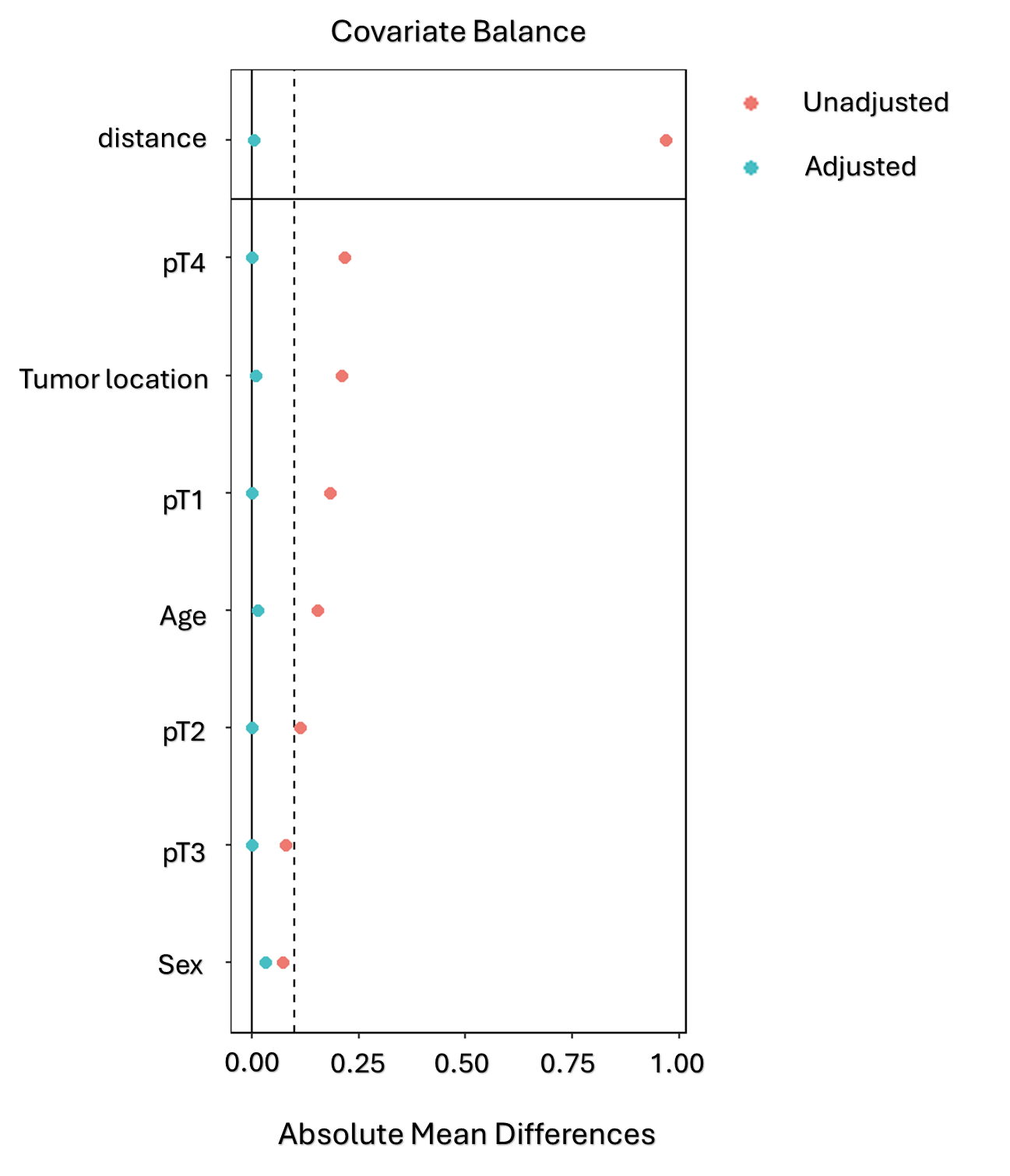

Supplement: Supplementary file 1 — Figure S1: Covariate balance before and after propensity score matching. Absolute mean differences in baseline characteristics between the perforated colorectal cancer and nonperforated colorectal cancer groups are shown before (red) and after (blue) PSM. The vertical dashed line represents a standardized difference of 0.1, indicating an acceptable balance. pT, pathological T stage; PSM, propensity score matching. [file AGS3-10-779-s003.docx]
