# Supplementary material for: Independent Prognostic Significance of Perforation in Colorectal Cancer: Insights From a Propensity Score‐Matched Cohort Study
Source: Ann Gastroenterol Surg. 2025 Dec 29;10(3):779–91. doi: 10.1002/ags3.70163 (PMC13178268; doi:10.1002/ags3.70163)

Supplementary Figure. 2 Recurrence-free survival (RFS) and overall survival (OS) according to adjuvant chemotherapy (AC) status in the AC-eligible cohort


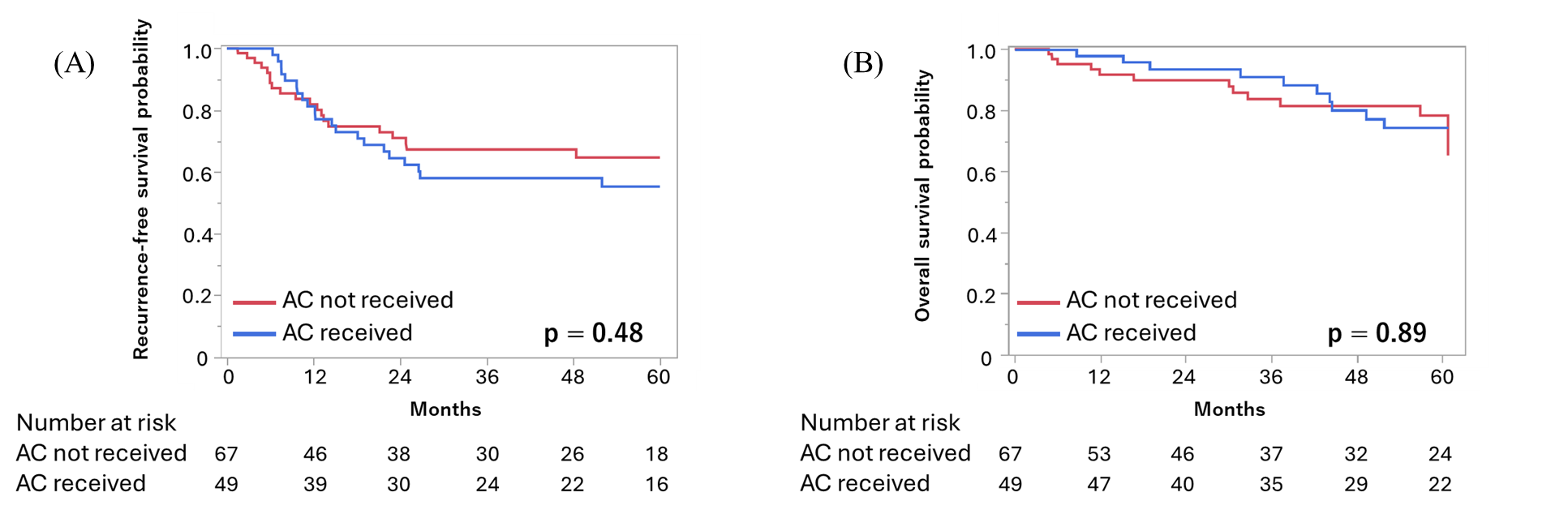

Supplement: Supplementary file 2 — Figure S2: Recurrence‐free survival (RFS) and overall survival (OS) according to adjuvant chemotherapy (AC) status in the AC‐eligible cohort. (A) RFS did not differ significantly between patients who received AC and those who did not (log‐rank p = 0.48). (B) OS likewise showed no significant difference between the groups (log‐rank p = 0.89). [file AGS3-10-779-s005.docx]
