# Supplementary material for: Independent Prognostic Significance of Perforation in Colorectal Cancer: Insights From a Propensity Score‐Matched Cohort Study
Source: Ann Gastroenterol Surg. 2025 Dec 29;10(3):779–91. doi: 10.1002/ags3.70163 (PMC13178268; doi:10.1002/ags3.70163)

Supplementary Figure 3. Hazard ratios (HRs) for recurrence-free survival (RFS) and overall survival (OS) associated with perforation, stratified by surgical era.


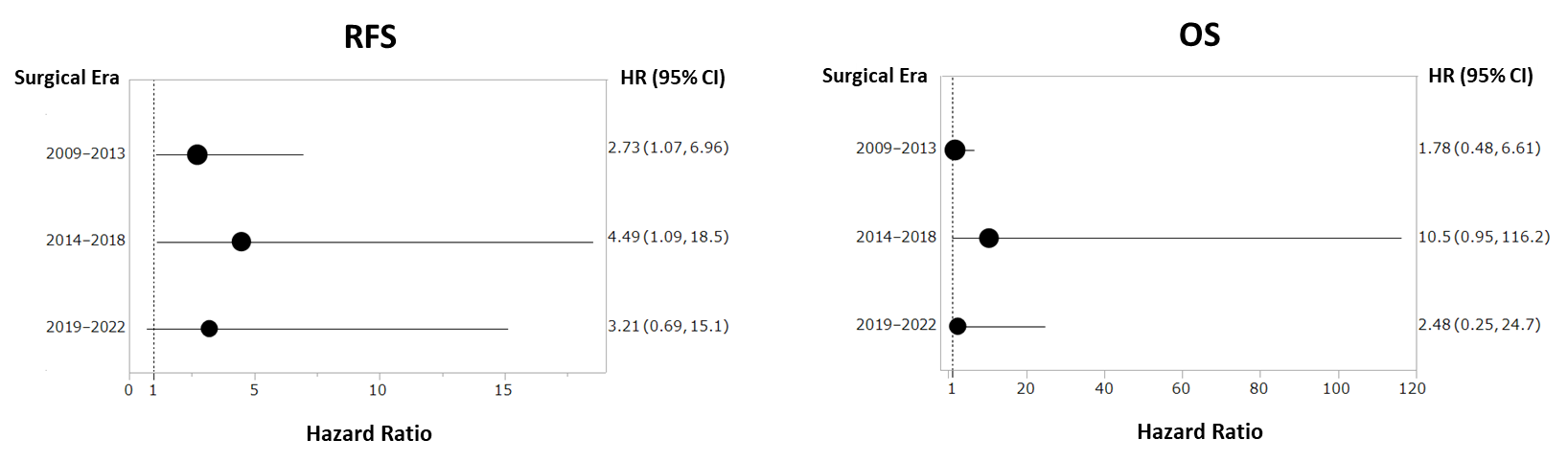

Supplement: Supplementary file 3 — Figure S3: Hazard ratios (HRs) for recurrence‐free survival (RFS) and overall survival (OS) associated with perforation, stratified by surgical era. Cox proportional hazards models were fitted separately for each era (2009–2013, 2014–2018, and 2019–2022) to estimate HRs associated with perforation. Dots represent HRs, and horizontal lines denote 95% confidence intervals. The vertical dashed line indicates no effect (HR = 1). [file AGS3-10-779-s006.docx]
