# Supplementary material for: Independent Prognostic Significance of Perforation in Colorectal Cancer: Insights From a Propensity Score‐Matched Cohort Study
Source: Ann Gastroenterol Surg. 2025 Dec 29;10(3):779–91. doi: 10.1002/ags3.70163 (PMC13178268; doi:10.1002/ags3.70163)
Supplement: Supplementary file 4 — Table S1: Adjuvant Chemotherapy (AC) administration in the AC‐eligible cohort. [file AGS3-10-779-s007.docx]

|  | **Supplementary Table. 1　 Adjuvant Chemotherapy(AC) Administration in the AC-Eligible Cohort** | | | | |  |  |
| --- | --- | --- | --- | --- | --- | --- | --- |
|  |  |  |  |  |  |  |  |
|  |  |  | **PCC (n = 48)** | **NPCC (n = 68)** | **P-value** |  |  |
|  | **Adjuvant chemotherapy** | |  |  |  |  |  |
|  |  | **Received** | **20 (41.7%)** | **29 (42.6%)** | **0.92** |  |  |
|  |  | **Not received** | **28 (58.3%)** | **39 (57.4%)** |  |  |  |
|  | **Interval to adjuvant chemotherapy (days)** | | **33.5 (29–46.5)** | **34 (25–45)** | **0.5** |  |  |
|  | **Adjuvant chemotherapy regimen** | |  |  | **0.85** |  |  |
|  |  | **Fluoropyrimidine monotherapy** | **14 (29.2%)** | **21 (30.9%)** |  |  |  |
|  |  | **Oxaliplatin-containing regimen** | **6 (12.5%)** | **8 (11.8%)** |  |  |  |
|  | **Initial recurrence pattern** | |  |  |  |  |  |
|  |  | **Distant metastasis** | **7 (14.6%)** | **8 (11.8%)** | **0.58** |  |  |
|  |  | **Peritoneal dissemination** | **3 (6.2%)** | **5 (7.4%)** | **0.83** |  |  |
|  |  | **Local recurrence** | **0** | **0** | **-** |  |  |
|  |  |  |  |  |  |  |  |
|  |  |  |  |  |  |  |  |
| MIS, minimally invasive surgery; PCC, perforated colorectal cancer; NPCC, non-perforated colorectal cancer. Data are presented as median (interquartile range) or number (%). | | | | | | | |
|  |  |  |  |  |  |  |  |
