# Supplementary material for: Independent Prognostic Significance of Perforation in Colorectal Cancer: Insights From a Propensity Score‐Matched Cohort Study
Source: Ann Gastroenterol Surg. 2025 Dec 29;10(3):779–91. doi: 10.1002/ags3.70163 (PMC13178268; doi:10.1002/ags3.70163)
Supplement: Supplementary file 5 — Table S2: Independent predictors of recurrence‐free survival in Multivariate Cox regression analysis in the AC‐eligible cohort. [file AGS3-10-779-s004.docx]

| **Supplementary Table.2　 Independent Predictors of Recurrence-Free Survival in Multivariate Cox Regression Analysis in the AC-Eligible Cohort** | | | | | | |
| --- | --- | --- | --- | --- | --- | --- |
|  |  |  |  |  |  |  |
|  |  |  | **Multivariate** | | |  |
|  |  |  | **HR** | **95 % CI** | **P-value** |  |
| **T stage** | **pT1–3 (ref)** |  | **1** | **—** | **—** |  |
|  | **pT4** |  | **2.73** | **1.4–5.33** | **0.0032** |  |
| **pN** | **pN0 (ref)** |  | **1** | **—** | **—** |  |
|  | **pN1–2** |  | **2.09** | **0.96–4.55** | **0.064** |  |
| **Perforation** | **Absent (ref)** |  | **1** | **—** | **—** |  |
|  | **Present** |  | **2.43** | **1.3–4.54** | **0.0052** |  |
| **Lymphatic invasion** | **Absent (ref)** |  | **1** | **—** | **—** |  |
|  | **Present** |  | **1.29** | **0.58–2.9** | **0.53** |  |
| **Adjuvant chemotherapy** | **Not received (ref)** |  | **1** | **—** | **—** |  |
|  | **Received** |  | **0.66** | **0.33–1.33** | **0.24** |  |
|  |  |  |  |  |  |  |
|  |  |  |  |  |  |  |
| HR, hazard ratio; CI, confidence interval; Ref, reference category | | | | | | |
|  |  |  |  |  |  |  |
