# Supplementary material for: Independent Prognostic Significance of Perforation in Colorectal Cancer: Insights From a Propensity Score‐Matched Cohort Study
Source: Ann Gastroenterol Surg. 2025 Dec 29;10(3):779–91. doi: 10.1002/ags3.70163 (PMC13178268; doi:10.1002/ags3.70163)
Supplement: Supplementary file 6 — Table S3: Independent predictors of overall survival in Multivariate Cox regression analysis in the AC‐eligible cohort. [file AGS3-10-779-s008.docx]

| **Supplementary Table.3 Independent Predictors of Overall Survival in Multivariate Cox Regression Analysis in the AC-Eligible Cohort** | | | | | | |
| --- | --- | --- | --- | --- | --- | --- |
|  |  |  |  |  |  |  |
|  |  | **Multivariate** | | |  |  |
|  |  | **HR** | **95 % CI** | **P-value** |  |  |
| **pT** | **pT1–3 (ref)** | **1** | **—** | **—** |  |  |
|  | **pT4** | **2.11** | **0.86–5.14** | **0.1** |  |  |
| **Perforation** | **Absent (ref)** | **1** | **—** | **—** |  |  |
|  | **Present** | **2.38** | **0.96–5.88** | **0.06** |  |  |
| **Postoperative complication** | **CD ≤ II (ref)** | **1** | **—** | **—** |  |  |
|  | **CD ≥ III** | **2.66** | **0.94–7.5** | **0.065** |  |  |
| **Adjuvant chemotherapy** | **Not received (ref)** | **1** | **—** | **—** |  |  |
|  | **Received** | **0.77** | **0.32–1.83** | **0.55** |  |  |
|  |  |  |  |  |  |  |
| HR, hazard ratio; CI, confidence interval; Ref, reference category; CD, Clavien–Dindo classification | | | | | |  |
