# Supplementary material for: Independent Prognostic Significance of Perforation in Colorectal Cancer: Insights From a Propensity Score‐Matched Cohort Study
Source: Ann Gastroenterol Surg. 2025 Dec 29;10(3):779–91. doi: 10.1002/ags3.70163 (PMC13178268; doi:10.1002/ags3.70163)
Supplement: Supplementary file 7 — Table S4: Multivariate Cox regression analysis for recurrence‐free survival adjusted for Surgical Era. [file AGS3-10-779-s001.docx]

| **Supplementary Table.4　 Multivariate Cox Regression Analysis for Recurrence-Free Survival Adjusted for Surgical Era** | | | | | | | | |
| --- | --- | --- | --- | --- | --- | --- | --- | --- |
|  |  |  |  |  |  |  |  |  |
|  |  |  | **Multivariate** | | |  |  |  |
|  |  |  | **HR** | **95 % CI** | **P-value** |  |  |  |
| **T stage** | **pT1–3 (ref)** |  | **1** | **—** | **—** |  |  |  |
|  | **pT4** |  | **2.61** | **1.35–5.03** | **0.0042** |  |  |  |
| **pN** | **pN0 (ref)** |  | **1** | **—** | **—** |  |  |  |
|  | **pN1–2** |  | **2.94** | **2.35-6.41** | **0.0068** |  |  |  |
| **Perforation** | **Absent (ref)** |  | **1** | **—** | **—** |  |  |  |
|  | **Present** |  | **2.77** | **1.43–5.38** | **0.0026** |  |  |  |
| **Lymphatic invasion** | **Absent (ref)** |  | **1** | **—** | **—** |  |  |  |
|  | **Present** |  | **1.18** | **0.51–2.71** | **0.7** |  |  |  |
| **Adjuvant chemotherapy** | **Not received (ref)** |  | **1** | **—** | **—** |  |  |  |
|  | **Received** |  | **0.66** | **0.34–1.3** | **0.23** |  |  |  |
| **Surgical era** | **2014–2018 vs 2009–2013** | | **0.56** | **0.26–1.24** | **0.15** |  |  |  |
|  | **2019–2022 vs 2009–2013** | | **1.48** | **0.66–3.32** | **0.34** |  |  |  |
|  | **2019–2022 vs 2014–2018** | | **2.64** | **1.04–6.7** | **0.04** |  |  |  |
|  |  |  |  |  |  |  |  |  |
| HR, hazard ratio; CI, confidence interval; Ref, reference category | | | |  |  |  |  |  |
