# Supplementary material for: Independent Prognostic Significance of Perforation in Colorectal Cancer: Insights From a Propensity Score‐Matched Cohort Study
Source: Ann Gastroenterol Surg. 2025 Dec 29;10(3):779–91. doi: 10.1002/ags3.70163 (PMC13178268; doi:10.1002/ags3.70163)
Supplement: Supplementary file 8 — Table S5: Multivariate Cox regression analysis for overall survival adjusted for Surgical Era. [file AGS3-10-779-s002.docx]

| **Supplementary Table.5 Multivariate Cox Regression Analysis for Overall Survival Adjusted for Surgical Era** | | | | | | |
| --- | --- | --- | --- | --- | --- | --- |
|  |  |  |  |  |  |  |
|  |  | **Multivariate** | | |  |  |
|  |  | **HR** | **95 % CI** | **P-value** |  |  |
| **pT** | **pT1–3 (ref)** | **1** | **—** | **—** |  |  |
|  | **pT4** | **2.28** | **0.93–5.61** | **0.07** |  |  |
| **Perforation** | **Absent (ref)** | **1** | **—** | **—** |  |  |
|  | **Present** | **3.36** | **1.31–8.54** | **0.011** |  |  |
| **Postoperative complication** | **CD ≤ II (ref)** | **1** | **—** | **—** |  |  |
|  | **CD ≥ III** | **2.73** | **0.9–8.3** | **0.076** |  |  |
| **Adjuvant chemotherapy** | **Not received (ref)** | **1** | **—** | **—** |  |  |
|  | **Received** | **0.9** | **0.37–2.19** | **0.81** |  |  |
| **Surgical era** | **2014–2018 vs 2009–2013** | **0.5** | **0.16-1.51** | **0.22** |  |  |
|  | **2019–2022 vs 2009–2013** | **1.16** | **0.36-3.77** | **0.81** |  |  |
|  | **2019–2022 vs 2014–2018** | **2.33** | **0.65-8.44** | **0.2** |  |  |
|  |  |  |  |  |  |  |
| HR, hazard ratio; CI, confidence interval; Ref, reference category; CD, Clavien–Dindo classification | | | | | |  |
